# Increased Chromosome Aberrations in Cells Exposed Simultaneously to Simulated Microgravity and Radiation

**DOI:** 10.3390/ijms20010043

**Published:** 2018-12-22

**Authors:** Megumi Hada, Hiroko Ikeda, Jordan R. Rhone, Andrew J. Beitman, Ianik Plante, Hikaru Souda, Yukari Yoshida, Kathryn D. Held, Keigi Fujiwara, Premkumar B. Saganti, Akihisa Takahashi

**Affiliations:** 1 Radiation Institute for Science & Engineering, Prairie View A&M University, Prairie View, TX 77446, USA; jrrhone@pvamu.edu (J.R.R.); ajbeitman@pvamu.edu (A.J.B.); Pbsaganti@pvamu.edu (P.B.S.); 2 Gunma University Initiative for Advanced Research, Gunma University, Maebashi, Gunma 371-8511, Japan; hi-ikeda@gunma-u.ac.jp (H.I.); kheld@mgh.harvard.edu (K.D.H.); 3 KBRwyle, 2400 NASA Parkway, Houston, TX 77508, USA; ianik.plante-1@nasa.gov; 4 Heavy Ion Medical Center, Gunma University, Maebashi, Gunma 371-8511, Japan; souda@gunma-u.ac.jp (H.S.); yyukari@gunma-u.ac.jp (Y.Y.); a-takahashi@gunma-u.ac.jp (A.T.); 5 Department of Radiation Oncology, Massachusetts General Hospital/Harvard Medical School, Boston, MA 02114, USA; 6 Department of Cadiology, University of Texas MD Anderson Cancer Center, Houston, TX 77030, USA; KFujiwara1@mdanderson.org

**Keywords:** microgravity, ionizing radiation, space radiation, chromosome aberration

## Abstract

Space radiation and microgravity (μ*G*) are two major environmental stressors for humans in space travel. One of the fundamental questions in space biology research is whether the combined effects of μ*G* and exposure to cosmic radiation are interactive. While studies addressing this question have been carried out for half a century in space or using simulated μ*G* on the ground, the reported results are ambiguous. For the assessment and management of human health risks in future Moon and Mars missions, it is necessary to obtain more basic data on the molecular and cellular responses to the combined effects of radiation and µ*G.* Recently we incorporated a μ*G*–irradiation system consisting of a 3D clinostat synchronized to a carbon-ion or X-ray irradiation system. Our new experimental setup allows us to avoid stopping clinostat rotation during irradiation, which was required in all other previous experiments. Using this system, human fibroblasts were exposed to X-rays or carbon ions under the simulated μ*G* condition, and chromosomes were collected with the premature chromosome condensation method in the first mitosis. Chromosome aberrations (CA) were quantified by the 3-color fluorescent in situ hybridization (FISH) method. Cells exposed to irradiation under the simulated μ*G* condition showed a higher frequency of both simple and complex types of CA compared to cells irradiated under the static condition by either X-rays or carbon ions.

## 1. Introduction

A long-duration exploration mission, such as a mission to Mars, will require humans to live in space for up to 3 years. All living organisms undergo physiological changes in response to the space environment—microgravity (µ*G*) in particular. It is well known that exposure to the µ*G* environment causes a range of detrimental health effects on astronauts, including bone and muscle loss, cardiovascular deconditioning, and neurovestibular changes [1,2]. Some of these health effects have been observed in studies using ground-based analogs that simulate the µ*G* condition [3].

The effects of gravity and microgravity on mammalian cells were known long before gravity effects on humans because cells can be exposed, relatively easily, to hypergravity by centrifugation and to simulated microgravity by using clinostats. Voluminous data on microgravity effects on cultured mammalian cells are now available from ground-based experiments using clinostats and also from experiments carried out on the International Space Station [4,5,6]. Some major changes noted in cultured mammalian cells when they are placed in a simulated [4,5,6] or true [7] microgravity environment include proliferation, motility, cytoskeletal reorganization, substrate adhesion, extracellular matrix synthesis, Ca^2+^ homeostasis, gene and protein expressions, and cell signaling. These changes are observed in many cell types, but the direction of changes (i.e., increase or decrease), such as for proliferation and motility, appears to depend on the cell type.

In addition to microgravity, cosmic radiation is another challenging factor in spaceflight. Deep space radiation risks arise primarily from solar energetic particles (SEPs) and galactic cosmic rays (GCR). Cosmic rays consist of approximately 85% protons and 14% helium ions (α particles), with the remaining 1% consisting of high-atomic-number and -energy (HZE) nuclei particles. During future missions back to the Moon or to Mars, astronauts will be exposed constantly to GCR and occasionally to particles from large solar particle events (SPE). Because the energy of some GCR particles is so high, it is difficult to shield the astronauts using conventional materials [8]. During a long-term deep space mission, astronauts will be exposed to 1–2 mSv/day of radiation and approximately half this value in interplanetary space and on planetary surfaces [9,10,11]. This amounts to a total estimated mission dose equivalent of ~1.01 Sv for a round trip to Mars consisting of 180 days of spaceflight (each way) and a stay of 500 days on the Martian surface during a particular solar cycle. Even though the flux levels of GCR particles are very low, the high-linear-energy-transfer (LET) particles produce dense ionization patterns as they pass through matter, so they have the ability to cause extensive damage to biomolecules.

Space radiation exposure may lead to an increase in cancer risk [12,13], degeneration of tissues such as cataract in the eye lens [14,15], and deleterious effects on the central nervous system such as reduced cognitive function and general neurological capacities [16]. At the cellular level, radiation induces DNA damage that needs to be fixed by the cellular repair mechanism. To counteract the potentially deleterious effects of DNA damage, cells activate the DNA damage response pathways which detect and repair DNA lesions. The specific repair pathway used to repair DNA depends on the types and complexity of the damage, and may not repair it properly. In addition to activating the DNA repair response, DNA damage also activates other signaling pathways such as those involved in cell cycle regulation and apoptosis.

Although space radiation and µ*G* are two major environmental stressors encountered simultaneously during space travel, our understanding of the combined effects of these two space conditions on cells and organisms is limited. Nevertheless, there is a limited number of studies to assess whether simultaneous exposure of cells and organisms to µ*G* and space radiation produces additive or synergistic consequences using a number of biological endpoints such as DNA damage response [17] in the International Space Station (ISS). Various organisms were pre-irradiated before space flight to test the effect of µ*G* on the repair of radiation-induced DNA damage. The results are controversial. In some cases, no significant effects were found [18,19,20], while in some other studies, either enhanced effects [21,22] or suppressed effects [23] were noted. Although the reason for these variable results is not known, it is possible that they may be due to the diversity of the biological systems used for these studies under various different experimental conditions. For the assessment and management of human health risks in future Moon and Mars missions, it is necessary to obtain more data on the molecular and cellular responses to combined effects of radiation under µ*G.*

In ground-based studies, two-dimensional rotators or 3-dimensional (3D) clinostats have been used to simulate μ*G*. These devices can create a simulated gravitational environment whose time-averaged gravitational vector size becomes extremely small, hence μ*G*. This condition is achieved by constantly and multidirectionally rotating samples [24]. However, problems arise when cells need to be irradiated at the same time. To perform the irradiation, it was necessary to stop the rotating device during the irradiation [25,26]. Stopping and restarting rotation may exert an additional gravitational stimulus on cells and may activate certain signaling cascades. Indeed, in our preliminary studies with endothelial cells*,* phosphorylation of several proteins was observed within 10–15 min after gravity changes, indicating that cells are able to respond to a new gravity condition rather quickly (Fujiwara, unpublished observation). Our results suggest that the experiments done by stopping and restarting clinostats would activate certain signaling events independently of radiation exposure. Thus, in order to understand the combined effects of μ*G* and space radiation, it is important to keep the same μ*G* condition before, during, and after exposure to radiation. In addition to these technical difficulties, the radiation types reported in most of these published studies are not comparable to deep space radiation in either quality or quantity [17].

In order to solve these problems, we recently developed systems by combining a 3D clinostat with synchronized irradiation systems (heavy-ion beam and X-ray) so that mammalian cells can be irradiated without stopping the rotation of the clinostat. In brief, this device is a 3D clinostat whose sample stage faces the direction of the irradiating beam at the time of pulse irradiation (0.2 s) of samples. This is achieved by synchronizing the heavy-ion irradiation with the position and orientation of the sample platform in the path of the irradiating beam. This apparatus is available at the Gunma University Heavy Ion Medical Center (GHMC) and has been described in detail by Ikeda et al. [27]. The irradiation is performed by a respiratory gating system used for heavy-ion radiotherapy [28]. The 3D clinostat controller enables beam irradiation by sending a signal to the gating system of the generator in synchrony with the correct orientation of the clinostat sample stage. We have also developed a 3D clinostat similarly synchronized to an X-ray irradiation system with a high-speed shutter [29]. Cells were cultured in an enclosed irradiation chamber [30,31] using a CO_2_-independent medium. The chamber was mounted onto the simulated μ*G* apparatus and kept at 37 °C for the duration of the entire experiment. These devices allow us to study the combined effects of either high- or low-LET radiations on mammalian cells that are continuously exposed to simulated μ*G*.

Chromosomal aberrations have been shown to increase in the lymphocytes of astronauts after long-duration missions of several months in space [32,33,34]. Chromosome exchanges, especially translocations, are positively correlated with many types of cancers, and are therefore a potential biomarker of cancer risk associated with radiation exposure [33,34,35,36]. In fact, the relative biological effectiveness (RBE) factors for chromosomal aberrations are similar to the RBEs observed for induction of solid tumors [35,37,38]. Fluorescence in situ hybridization (FISH) chromosome painting methods for the analysis of chromosome aberrations can provide more insight into the complexity of the damage induced by radiations. The results of FISH painting studies indicate that high-LET radiation induces a much higher frequency of complex chromosome damages than does low-LET radiation, and the rearrangements are of greater complexity [39,40,41,42,43]. Therefore, chromosomal aberrations are a useful biomarker for cancer risks and for comparisons with other biomarkers in the absence of human data for galactic cosmic ray effects [8,44]. In this study, we irradiated human fibroblasts (1BR-hTERT) under simulated microgravity with C-ions and X-rays and assessed the formation of chromosome aberrations.

## 2. Results

### 2.1. Cell Survival

**Figure 1:**
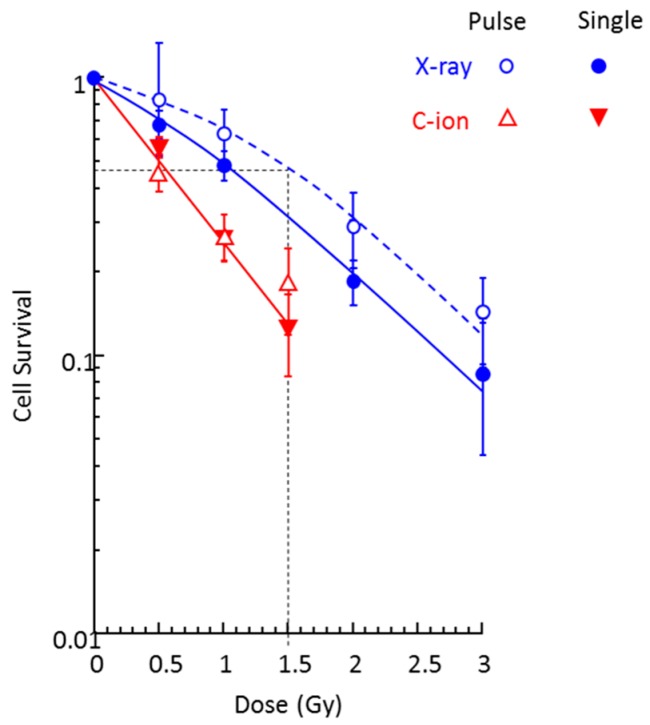
Survival curves of human fibroblasts irradiated by a single dose (open symbols) or the same cumulative dose given by 0.2 s pulses (closed symbols) of X-ray and 290 MeV/n C-ion beam under static conditions. Experimental data represent the mean of two plates from four experiments.

Figure 1 shows the survival curves of fibroblasts irradiated by X-rays and 290 MeV/n C-ions under the static condition. Since cells were exposed with 0.2 s pulses under simulated μ*G* conditions, the survival study was conducted with 0.2 s pulse exposure as well as continuous exposure. For each dose level, the cumulative dose level of pulse irradiation was the same as the dose by continuous exposure. Compared to typical survival curves with a shoulder in the lower dose ranges with X-rays, exponential survival curves for C-ions show no apparent shoulder. The survival curve was similar for both pulse and continuous exposures by C-ion beam. On the other hand, continuous X-ray exposure was more damaging to cells than pulse exposure. C-ions are more effective for cell killing compared to X-rays per unit dose.

The lethal dose 50 (LD50) value for X-ray pulse irradiation was 1.5 Gy, which is equivalent to 0.5 Gy C-ions exposure. Based on these results, we selected the doses of 0.5 Gy for C-ions and 0.5 and 1.5 Gy for X-rays to assess the combined effect of simulated μ*G* and radiation.

### 2.2. Chromosome Aberrations

**Figure 2:**
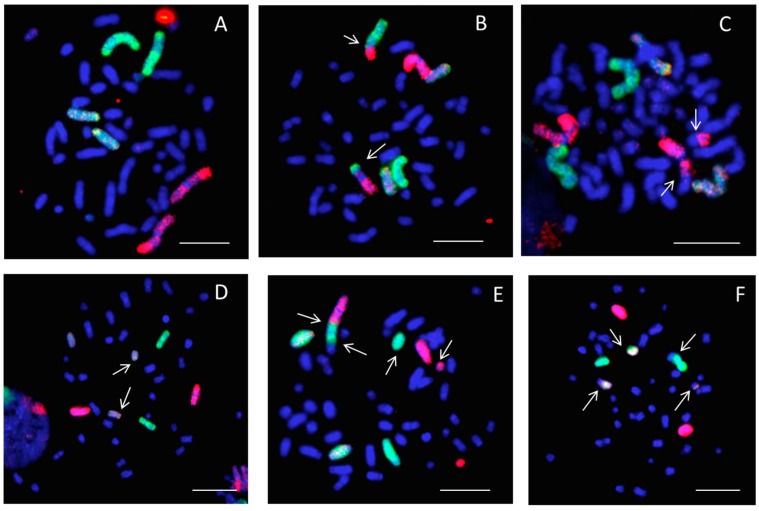
Examples of chromosome painting in human fibroblasts (1BR-hTERT) with 3-color whole-chromosome FISH: chromosome 1 (red), chromosome 2 (green), and chromosome 4 (yellow). Chromosome aberrations were identified by arrows as simple (reciprocal exchanges between two chromosomes) or complex-type exchanges (exchanges involving a minimum of three breaks in two or more chromosomes). **A**: normal; **B**: simple exchange between chromosome 1 and 2; **C**: simple exchange between chromosome 1 and other chromosome (dicentric); **D**: break in chromosome 4 complex; **E**: complex exchange in chromosomes 1 and 2 and another chromosome; **F**: complex exchange in chromosomes 2 and 4 and another chromosome). The scale bars represent 10 μm.

**Figure 3:**
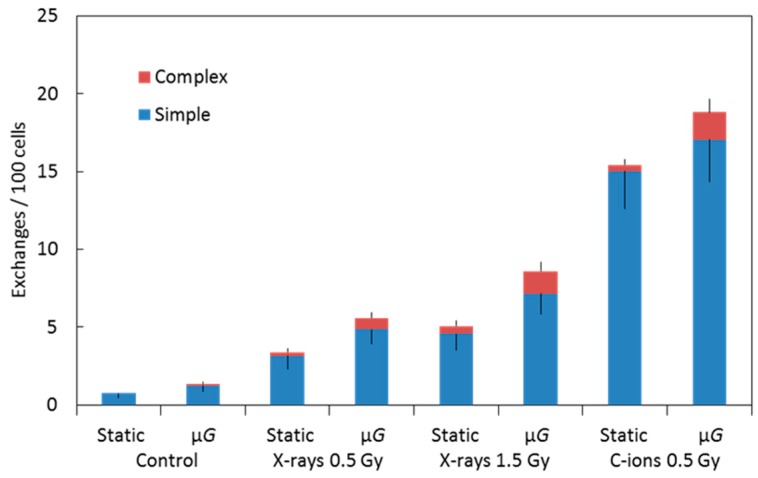
Graphic representation of the data shown in Table 1. Frequencies of simple and complex types of chromosome exchanges induced by X-ray or C-ion beam while cells were under either static or simulated μ*G* conditions. Error bars indicate the standard error of the mean values.

Figure 2 shows images of 3-color chromosome FISH in which chromosome 1 (red), chromosome 2 (green), and chromosome 4 (yellow) are identified. All chromosomes were labeled by DAPI (4,6-diamidino-2-phenylindole). Undamaged chromosomes are shown in Figure 2A. Other panels show chromosomes that are damaged in various ways (see figure legend). All types of detectable aberrations in chromosomes 1, 2, and 4 were scored, and the whole-genome equivalent frequencies of aberrations were calculated. Table 1 and Figure 3 show the frequencies of simple, complex, and total exchanges induced by X-ray and C-ion exposure under static and simulated μ*G* conditions that were measured in premature chromosome condensation (PCC) collected at the first division after exposure (Figure 2). The frequencies of background CA in cells exposed to the static or simulated μ*G* condition are average frequencies of several experiments on unirradiated cells. Simulated μ*G* alone increased the background CA frequencies for the total, simple, and complex exchanges. With 0.5 Gy or 1.5 Gy X-ray and 0.5 Gy C-ion, the frequencies of both simple and complex exchanges were increased under the simulated μ*G* condition compared to the static condition. Although 0.5 Gy of C-ion exposure and 1.5 Gy of X-ray exposure gave the same extent of cell survival, the level of chromosomal damage by 0.5 Gy C-ion was 2–3 times higher than 1.5 Gy X-ray. Our results suggest that the combined effects of μ*G* and exposure to cosmic radiation are interactive in causing chromosome aberrations in human fibroblasts.

### 2.3. Simulation of Track Structure of Irradiation

**Figure 4:**
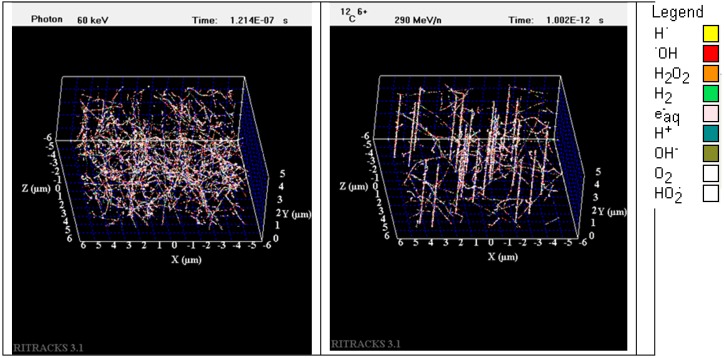
Simulation of the radiation track structures in a volume of 10 × 10 × 5 µm^3^ irradiated by 50 cGy of X-ray (**left**) and 50 cGy of carbon ion, 290 MeV/n (**right**). Each dot represents a radiolytic species (see legend for color code).

The experimental data show that both the survival and the extent of chromosome aberrations depend on the type of radiation to which cells are exposed. At the scale of cells, which is roughly 50 × 50 × 15 µm^3^, the track structures of radiation used for this study differ greatly from one type to another. The radiation track structure may provide insights into understanding the different biological effects of radiation types. Figure 4 illustrates the radiation track structure within an irradiated volume of 10 × 10 × 5 µm^3^ by X-rays (The energy of the photons for this simulation is 60 keV. This energy corresponds to the characteristic energy emissions of X-ray sources. In reality, a bremsstrahlung component should be added to the energy spectra for most X-ray sources. Nevertheless, using the energy distribution from the spectra is not so important for this simulation as the characteristic energy emission is largely dominating. Furthermore, the contributions of bremsstrahlung photons would be to generate more electron tracks with similar energies to the irradiated volume) and 290 MeV/n C-ions simulated by the software RITRACKS (Relativistic Ion Tracks). This volume is roughly the size of the nucleus of a fibroblast. The dose to the volume is approximately 0.5 Gy in both cases. This dose was chosen to better illustrate the difference between irradiation by X-rays and C-ion beam. In Figure 4, on the left, electron tracks corresponding to Compton and photoelectrons are observed. On the right, 24 C-ion tracks are shown. The track cores, which are the linear structures, are clearly seen. The LET of the C-ions used for the simulation was 12.9 keV/μm. Although the irradiated volume received the same dose in both cases, the track structures and the pattern of energy deposition are quite different. Carbon ions and other ions in general have typical track structures composed by a track core, which is mostly linear and comprising dense ionizations, and a penumbra, which is composed by tracks generated by the secondary electrons. On the other hand, photons have large mean free paths (between two interactions) and are deflected with large angles. Therefore, Compton electrons and photoelectrons appear in random locations and directions in the irradiated volume.

## 3. Discussion

The interplay between radiation and simulated microgravity on CA is controversial. Manti et al. reported no effect of simulated μ*G* created by a rotating wall vessel in human lymphocytes irradiated by X-ray or proton beam [45]. However, Mosesso et al. showed increased CA in human lymphocytes exposed to 1.5 Gy of X-rays and μ*G* using a clinostat [46]. Their results are similar to our results obtained by using fibroblasts exposed to X-ray or C-ion beam on a clinostat. Increased mutation rates were also reported in human lymphoblastoids and lymphocytes in a rotating wall vessel and exposed to γ-rays or X-rays [47,48]. In all of these studies, cells were cultured under simulated microgravity conditions after they had been irradiated, albeit all at a relatively high dose rate. By contrast, our cells were treated simultaneously with simulated μ*G* and pulse irradiation, and the levels of irradiation were at a lower dose rate that better simulates the condition of deep space.

Increased CA in cells exposed simultaneously to simulated microgravity and radiation compared to cells exposed to radiation alone could be explained by (1) increased cellular sensitivity to radiation under the μ*G* condition and/or (2) decreased ability of cells to repair damaged DNA. It is known that high-LET-energy heavy-ion beams produce more double-strand breaks (DSB) in DNA per unit dose than low-LET radiation [49] and cause complex and irreparable clustered DNA damage [50]. Several studies suggest that non-DSB clustered lesions play an important role in chromosomal instability through their repair resistance [51,52,53]. Changes in chromatin conformation and chromatin–chromatin interactions in human epithelial cells under simulated microgravity and super-G environments were suggested by our previous study on the folding of chromatin during interphase [54]. Takata et al. reported that chromatin compaction protects genomic DNA from radiation damage [55]. In this study, since we adapted cells to the μ*G* condition 24 h before irradiation, the chromatin structure might have changed such that the susceptibility of chromatin to radiation might have increased, leading to more DNA damage. Several investigators have also reported that microgravity may influence the cytoskeleton structure [56,57], and changes in the cytoskeleton, which is known to be involved in cell signaling including mechanosignaling [58,59], might affect DNA repair efficiency [60,61,62].

As for radiation-induced DSB, CA frequency depends on the efficiency of the DNA damage repair process [63]. Decreased DNA repair capacity was reported by several researchers in human lymphocytes under simulated microgravity [64,65]. In our experiments, in order to allow cells to repair damaged DNA, we kept cells under the simulated μ*G* condition for an additional 24 h after irradiation. It is possible that the DNA repair process could have been affected by the simulated μ*G* condition. The control (non-irradiated) samples also showed increased frequency of CA with simulated μ*G*, possibly indicating μ*G*-induced downregulation of the DNA repair mechanism.

Compared to the low-LET (X-ray) exposure, the C-ion beam caused more chromosome aberrations per unit dose. To understand the differences in the yield of chromosome aberrations by C-ions and X-rays, it is useful to look at the track structure of these two radiation types. For ions such as carbon, energy deposition is highly heterogeneous, with a localized contribution along the trajectory of every particle and lateral diffusion of energetic electrons (i.e., δ-rays, the target atom electrons ionized by the incident HZE ion and emitted at high energy) many microns from the path of the ions. These particles are therefore densely ionizing along the primary track (i.e., the track followed by the incident heavy ion, the so-called core). Moreover, they are surrounded by a region (penumbra) comprising the high-energy electrons ejected by ions [66]. The density of the core and penumbra depends mostly on the charge and velocity (energy per nucleon) of the ion. Since we can assume that an interaction between radiation tracks and DNA is necessary to create a break, as the energy deposited in the volume is mostly concentrated in the core regions of the tracks, DNA breaks tend to be formed in clusters and are difficult to repair properly. As clustered DNA breaks are prone to improper rejoinings, this leads to the formation of chromosome aberrations. The situation is quite different for X-rays that interact mostly by Compton and photoelectric effects, which result in considerable deflection of the photon after an interaction and the creation of a large number of electron tracks in the medium. As previously reported [67], the dose voxels were distributed randomly and scattered uniformly within the volume irradiated by low-LET radiation (X-ray), whereas the rasterized image of the track structure could be seen for the carbon particles. As energy deposited by X-rays is mostly dispersed in the irradiated volume, DNA breaks are much less clustered, which makes them easier to repair properly.

## 4. Materials and Methods

### 4.1. Cell Culture

Human fibroblasts (1BR-hTERT cells) were kindly provided by Dr. P.A. Jeggo (Sussex University, Brighton, UK) and Dr. A. Shibata (Gunma University, Gunma, Japan). Cells were cultured in CO_2_-independent medium (COI) (Thermo Fischer Scientific, Waltham, MA, USA) supplemented with 10% (*v*/*v*) fetal bovine serum (MP Biomedicals, Santa Ana, CA, USA), 200 mM l-glutamine (Thermo Fischer Scientific), and penicillin–streptomycin mixed solution (Nacalai Tesque, Kyoto, Japan) at 37 °C. Exponentially growing cells were cultured in disposable, sealed irradiation cell culture chambers (Chiyoda Co., Kanagawa, Japan) [30,31] for 24 h after seeding, and the culture medium was then replaced with fresh COI medium before setting into the 3D clinostat (PMS-CST I, Advanced Engineering Services Co. Ltd (AES), Ibaraki, Japan) for simulated microgravity (μ*G*) or the static stage (AES) for 1*G* control as previously reported [27].

### 4.2. Synchronized Irradiation Systems under Simulated μG or 1G

The irradiation of cells without stopping the clinostat motion was achieved by 0.2 s of pulse irradiation when the cell growth surface of the chamber on the clinostat became perpendicular to the beam of irradiation. The controller of the 3D clinostat was also connected to a high-speed shutter system for X-ray irradiation or a respiratory gating system for C-ions irradiation to achieve this specific positioning (i.e., synchronization) of the chamber orientation and the timing of the pulse irradiation, which occurred every 60 s. Synchronized X-ray irradiation was performed using an X-ray generator (200 kV, 14.6 mA, aluminum filter (0.3 mm thick), MultiRad225: Faxitron Bioptics, LLC, Tucson, AZ, USA) equipped with a high-speed shutter (Accelerator Engineering Co. (AEC), Chiba, Japan). Synchronized C-ion irradiation was done using a synchrotron (Gunma University Heavy Ion Medical Center, Gunma, Japan) and respiratory gating signals with a dose-averaged linear energy transfer of 50 keV/μm at the center of the 6 cm spread-out Bragg peak (SOBP) of the beam with an energy of 290 MeV/n [68]. For the control, cells in the same chamber mounted on a stationary clinostat (1*G*) were pulse irradiated for 0.2 s every 60 s [27,29,68,69]. Doses ranged from 0.5 to 3 Gy and dose rates were 0.03 Gy/min for both X-ray and C-ion irradiation under the simulated μ*G* or 1*G* conditions.

### 4.3. Cell Survival Colony Formation Assay

1BR-hTERT cell survival was measured using a standard colony forming assay. T25 Falcon^®^ flasks (Corning Incorporated, New York, NY, USA) were employed, and four independent experiments were repeated for each irradiation dose. Colonies formed after 14 days of irradiation were fixed with methanol and stained with 5% Giemsa solution. Colonies composed of more than approximately 50 cells were counted as surviving cells and scored.

### 4.4. Premature Chromosome Condensation (PCC)

The PCC technique was used to collect G2/M-phase chromosomes as previously described [63,70,71]. After irradiation, fibroblasts were allowed to recover for 24 h under static or μ*G* conditions and then subcultured at low density. After 33 h incubation, cells were arrested in mitosis by adding KaryoMAX^®^ Colcemid^®^ solution (Thermo Fisher Scientific) to a final concentration of 90 ng/mL in the culture media, and then cells were incubated for an additional 7 h. Approximately 30 min before collection, 50 nM of Calyculin A (FUJIFILM Wako Pure Chemical Co., Osaka, Japan) was added to the culture media to condense the chromosomes in the G2 phase of the cell cycle.

### 4.5. Fluorescence In Situ Hybridization (FISH)

Chromosome spreads were prepared as described [72] and were hybridized in situ with a combination of three fluorescence whole-chromosome human DNA probes for chromosomes 1 (red), 2 (green), and 4 (yellow) (Aquarius, Cytocell, Oxford Gene Technology, Oxfordshire, UK), using the protocol recommended by the manufacturer. All chromosomes were then stained with DAPI. Chromosomes were analyzed with the Leica Cytovision fluorescence in situ hybridization (FISH) system which includes a Leica fluorescent microscope with a charge-coupled device (CCD) camera and karyotyping software. Images of all cells with damaged chromosomes in chromosomes 1, 2, and 4 were captured electronically. Complex exchanges were scored when it was determined that an exchange involved a minimum of three breaks in two or more chromosomes [70]. An exchange was defined as simple if two breaks in two chromosomes were noted, that is, dicentrics and translocations. Incomplete translocations and incomplete dicentrics were included in the category of simple exchanges, assuming that in most cases the reciprocal fragments are below the level of detection. Each type of exchange (dicentrics, apparently simple reciprocal exchanges, incompletes, or complex) was counted as one exchange, and values for total exchanges were derived by adding the yields. When two or more painted chromosomes were damaged, each was scored separately. For each experiment consisting of a single beam at multiple doses, at least 500 cells were scored for each datapoint.

### 4.6. Simulations of Radiation Tracks

Simulation of the radiation track structures for both X-ray and C-ion beam was performed with the code RITRACKS (Relativistic Ion Tracks) developed at the NASA Johnson Space Center. RITRACKS simulates the primary interactions of the ions and photons with matter and calculates the energy of all secondary electrons produced in the medium. Because the secondary electrons will lead to further ionization of molecules in the medium, secondary electron tracks are also simulated. The detailed algorithms for this simulation code were described in Plante and Cucinotta [67] and in the references therein. For both irradiation beams, but more importantly for X-ray irradiation, periodic boundary conditions were applied to simulate the contribution of radiation from the neighboring volumes to the volume of interest.

### 4.7. Statistical Analysis

The frequencies of chromosomal aberrations in painted chromosomes were evaluated as the ratio between aberrations scored and total cells analyzed. Several studies have indicated that the distribution of radiation damage among chromosomes is random, and the yield of exchanges measured within the first division after exposure is proportional to the DNA content of the chromosome analyzed, with some fluctuation of data [73]. Therefore, the frequencies of exchanges in individual chromosomes can be extrapolated to whole-genome equivalents using a modified version of the Lucas et al. [74] formula, Fp = 2.05 [fp (1 − fp) + fp1 fp2 + fp1 fp3 + fp2 fp3] FG. FP is the combined frequency of exchanges in all painted chromosomes; fp is the fraction of the whole genome comprising the painted chromosomes; fp1, fp2, and fp3 are the fractions of the genome for each individual chromosome; and FG is the whole-genome aberration frequency. Using this formula, the genomic frequency for 1BR-hTERT fibroblasts was estimated as 2.48 times that detected in chromosomes 1, 2, and 4.

The standard errors for aberration frequencies were calculated assuming Poisson statistics. The error bars in figures represent the standard error of the mean values.

## 5. Conclusions

Our studies show that simultaneous exposure of human fibroblasts to simulated μ*G* and cosmic radiation results in greater frequency of CA than in cells exposed to radiation alone. A cancer risk assessment for space radiation based on the dose–response data from cells irradiated under static conditions might underestimate the potential risk for astronauts, as our findings show significantly increased CA frequency. We suggest that our findings may have important implications, requiring not only similar investigations on different cell types but also other end points and model systems to be investigated under the combined influence of μ*G* and irradiation.

## Figures and Tables

**Table 1:** Whole-genome equivalent for frequency of chromosome aberrations per 100 cells in human fibroblasts by X-ray and C-ion beam under static and simulated μ*G* conditions.

Radiation	Static or μ*G*	Total Spreads Scored	No. of Aberrant Spreads	Simple Exchanges	Complex Exchanges	Total Exchanges
Control (0 Gy)	Static	2025	13	0.73 ± 0.30	0	0.73 ± 0.30
	μ*G*	1994	19	1.24 ± 0.39	0.12 ± 0.12	1.37 ± 0.41
X-ray (0.5 Gy)	Static	1024	23	3.15 ± 0.87	0.24 ± 0.24	3.39 ± 0.91
	μ*G*	1376	12	4.86 ± 0.94	0.72 ± 0.36	5.58 ± 1.00
X-ray (1.5 Gy)	Static	1025	29	4.58 ± 1.05	0.48 ± 0.34	5.06 ± 1.10
	μ*G*	1038	38	7.16 ± 1.31	1.43 ± 0.58	8.59 ± 1.43
C-ions (0.5 Gy)	Static	643	50	15.03 ± 2.41	0.39 ± 0.39	15.42 ± 2.44
	μ*G*	566	48	17.07 ± 2.73	1.75 ± 0.88	18.83 ± 2.87

## Abbreviations

3D	3-Dimensional
CA	Chromosome aberrations
CCD	Charge-coupled device
COI	CO_2_-independent medium
DSB	Double-strand breaks
FISH	Fluorescence in situ hybridization
GCR	Galactic cosmic rays
GHMC	Gunma University Heavy Ion Medical Center
HZE	High atomic number and energy
ISS	International Space Station
LD50	Lethal dose 50
LET	Linear Energy Transfer
MeV/n	Mega electron Volt per nucleon
PCC	Premature chromosome condensation
SEP	Solar energetic particle
SE	Standard error
SPE	Solar particle events
SOBP	Spread-out Bragg peak
TAMU	Texas A&M University
µ*G*	Microgravity
